# Effect of Post-Reconstruction Gaussian Filtering on Image Quality and Myocardial Blood Flow Measurement with N-13 Ammonia PET

**Published:** 2014

**Authors:** Hyeon Sik Kim, Sang-Geon Cho, Ju Han Kim, Seong Young Kwon, Byeong-il Lee, Hee-Seung Bom

**Affiliations:** 1 Department of Nuclear Medicine, Chonnam National University Hwasun Hospital, Hwasun-Gun, Jeollanamdo, South Korea; 2 Department of Nuclear Medicine, Chonnam National University Hospital, Hwasun-Gun, Jeollanamdo, South Korea; 3 Department of Cardiology, Chonnam National University Hospital, Hwasun-Gun, Jeollanamdo, South Korea; 4 Korea Photonics Technology Institute, Gwangju City, South Korea

**Keywords:** Gaussian filtering, Myocardial blood flow, PET image reconstruction

## Abstract

**Objective(s):**

In order to evaluate the effect of post-reconstruction Gaussian filtering on image quality and myocardial blood flow (MBF) measurement by dynamic N-13 ammonia positron emission tomography (PET), we compared various reconstruction and filtering methods with image characteristics.

**Methods:**

Dynamic PET images of three patients with coronary artery disease (male-female ratio of 2:1; age: 57, 53, and 76 years) were reconstructed, using filtered back projection (FBP) and ordered subset expectation maximization (OSEM) methods. OSEM reconstruction consisted of OSEM_2I, OSEM_4I, and OSEM_6I with 2, 4, and 6 iterations, respectively. The images, reconstructed and filtered by Gaussian filters of 5, 10, and 15 mm, were obtained, as well as non-filtered images. Visual analysis of image quality (IQ) was performed using a 3-grade scoring system by 2 independent readers, blinded to the reconstruction and filtering methods of stress images. Then, signal-to-noise ratio (SNR) was calculated by noise and contrast recovery (CR). Stress and rest MBF and coronary flow reserve (CFR) were obtained for each method. IQ scores, stress and rest MBF, and CFR were compared between the methods, using Chi-square and Kruskal-Wallis tests.

**Results:**

In the visual analysis, IQ was significantly higher by 10 mm Gaussian filtering, compared to other sizes of filter (*P*<0.001 for both readers). However, no significant difference of IQ was found between FBP and various numbers of iteration in OSEM (*P*=0.923 and 0.855 for readers 1 and 2, respectively). SNR was significantly higher in 10 mm Gaussian filter. There was a significant difference in stress and rest MBF between several vascular territories. However CFR was not significantly different according to various filtering methods.

**Conclusion:**

Post-reconstruction Gaussian filtering with a filter size of 10 mm significantly enhances the IQ of N-13 ammonia PET-CT, without changing the results of CFR calculation.

## Introduction

Reconstruction and filtering of acquired images in myocardial perfusion imaging (MPI) are of extreme importance, since they are directly related to diagnostic accuracy. Image blurring, artifact, or image distortion can lead to false positive or negative results, which lead to wrong decision making regarding the treatment choice or prognostic stratification.

Image reconstruction and filtering harbor even more significant importance in cardiac positron emission tomography (PET) because it provides quantitative myocardial blood flow (MBF, ml/g/min) of tissue as well as coronary flow reserve (CFR) in addition to traditional relative tomographic images. Different reconstruction methods of PET images are directly correlated with different MBF results.

Generally, reconstruction methods are divided to analytic and iterative methods. Analytic methods include filtered back projection (FBP), Fourier rebinning (FORE), and three-dimensional reprojection (3DRP) algori-thms. Iterative approaches consist of ordered subsets expectation maximization (OSEM) and maximum likelihood expectation maximization (ML-EM).

The quality of OSEM images is superior to that of FBP images (1-3). FBP is a back-projected image after filtering of the sinogram. The noise of FBP image is less than that of back-projected (BP) image. Although the FORE method is developed for reducing the calculation time, it is not used considering its distorting effects on images.

ML-EM is an iterative image estimation method among the iterative approaches. Starting with an initial image guess, this method iteratively selects a new estimated image, based on the measured projections. If ML-EM method uses a total of subsets, OSEM method uses a subset of a total of subsets; therefore, OSEM method can reduce the calculation time.

OSEM method is developed for improving the disadvantages of ML-EM method (4). A disadvantage of this method is the longer processing time of iterative approaches, compared to the processing time of FBP method. However, iterative approaches can potentially increase the accuracy of images, compared to analytic approaches. The OSEM algorithm has been usually used for PET studies, due to noise reduction properties in regions of low uptake (5). However, it is established that noise increase is associated with an increasing number of iterations (6, 7).

Image filtering methods, including the popular Gaussian filtering, are used to reduce background noise and improve signal-to-noise ratio (SNR) of the image with better contrast (9). However, Gaussian filtering also generates image distortion, considering the size of filter, which makes the quantitative analysis of MBF difficult. The purpose of this study was to compare various combinations of FBP and OSEM with Gaussian filtering in the measurement of MBF, using ^13^N-NH_3_ dynamic PET, and to find an appropriate method.

## Methods

### Materials

^13^N-NH_3_ dynamic PET images were obtained from three patients with coronary artery disease (male-female ratio=2:1; age: 57, 53, and 76 years).

### Image acquisition

After CT transmission scan for an attenuation correction, 11 MBq/kg of ^13^N-NH_3_ was injected as a bolus (<5 s), followed by 6 min of dynamic image acquisition (12×5 s, 6×10 s, 3×20 s, 6×30 s) for rest MBF measurement. Thereafter, a 13-min gated image acquisition was performed. After an additional 50 min for the decay of ^13^N activity, pharmacologic stress was given by infusing adenosine (0.14 mg/kg/min) for 6 min. Stress imaging was performed immediately after the injection of 11 MBq/kg of ^13^N-NH_3,_ which was done at peak stress (3 min after the start of adenosine infusion). The stress image acquisition was done in the same method with that of the rest imaging.

### Image reconstruction and filtering

Acquired PET data were reconstructed by FBP and OSEM methods. In the FBP reconstruction method, the transaxial filter was set on enhanced Hanning, and the cutoff was set to 9.6 mm. In the OSEM reconstruction method, the subset was fixed to 21 and z axial filter was set to standard; Full Width at Half Maximum (FWHM) of post filter was fixed at 2.57 mm.

OSEM reconstruction method was divided into OSEM with two iterations (OSEM_2I), four iterations (OSEM_4I), and six iterations (OSEM_6I). Other factors of FBP and OSEM were set to default values. The reconstructed images were filtered by Gaussian filter, with sizes of 5, 10, and 15 mm.

### MBF measurement

The MBF of reconstructed images was measured by the cardiac pixel-wise modeling software (PMOD 3.204; University Hospital Zurich, Zurich, Switzerland). The heart model type and kinetic model were set to Human and Card NH3 (2 Compartments) [PK cardiac NH3 Hutchins Model].

“C_PET (t)=(1−V_lv−V_rv)(C_1 (t)+C_12 (t))+V_lv C_lv (t)+V_rv C_rv (t)”

Where V_lv_ and V_rv_ are spill-over fractions of blood activity in the left ventricle C_lv_(t) and right ventricle C_rv_(t).

We used the polar map of 17 segments; other factors were set to default values. The rest and stress PET images were automatically reoriented by the PMOD software. Finally, we could obtain the MBF for stress and rest and CFR of each segment, each territory: left anterior descending coronary artery (LAD), right coronary artery (RCA), left circumflex (LCX), and total myocardium.

### Image analysis

The visual image quality (IQ) was assessed by two experienced nuclear medicine phy-sicians, who were blinded to the reconstruction methods and filter size. Sixteen stress images, created by different reconstruction methods and filter sizes, were randomly rearranged and given to the two readers. Visual IQ was graded using a 3-grade scoring system, with 1, 2, and 3 representing poor, acceptable, and good quality, respectively. Total score was the sum of counted scores from each reader.

Signal-to-noise ratio (SNR) was calculated by noise and contrast recovery (CR). Noise was measured by standard deviation (σ_B_) and mean (μ_B_) in the right ventricle and CR was measured in myocardium. SNR was calculated by the equation of SNR=CR/(σ_B_/μ_B_) (10). We also calculated the perfusion ratios of normal to abnormal vascular territories like RCA/LAD and LCX/LAD using the polar map of CFR according to filtering methods.

### Statistical analysis

Image quality scores, stress and rest MBF, and CFR were compared between the methods, using Chi-square and Kruskal-Wallis tests.

## Results

**Figure 1:**
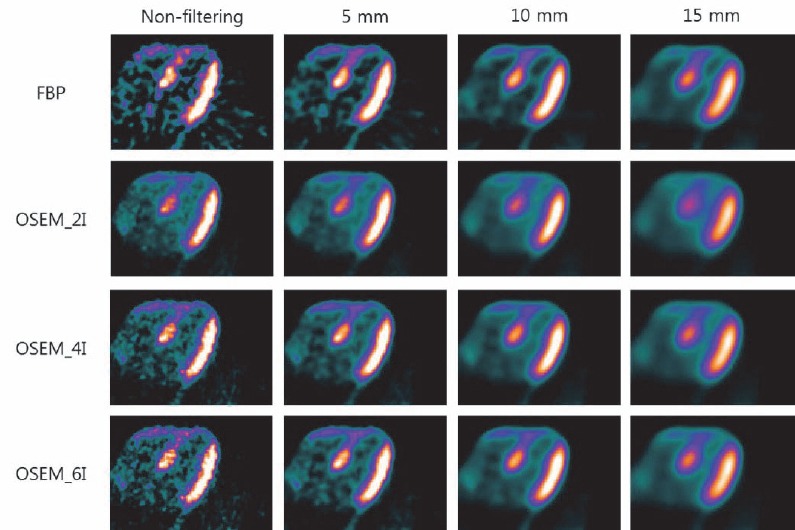
^13^N-NH_3_ cardiac PET image was reconstructed by FBP and OSEM. OSEM reconstruction method was divided into OSEM_2I, OSEM_4I, and OSEM_6I, since the number of iterations was set to 2, 4, and 6. The reconstructed images were filtered by 5, 10, and 15 mm Gaussian filters. The noise increased with an increasing number of iterations. The noise was decreased and blurring was intensified as the Gaussian filter size increases

**Figure 2:**
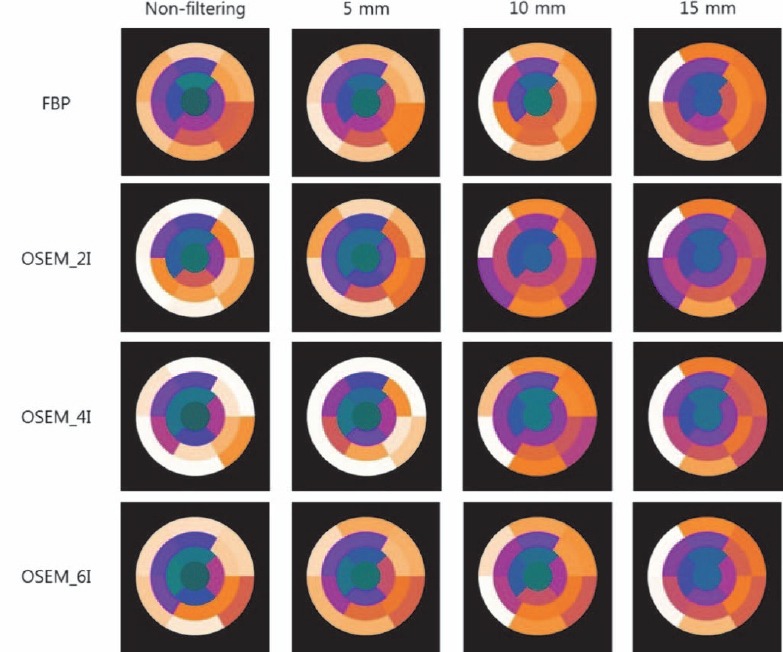
^13^N-NH_3_ cardiac PET image was reconstructed by FBP and OSEM (number of iterations: 2, 4, and 6) without filtering and with Gaussian filters (5, 10, and 15 mm). The polar maps of CFR were obtained by reconstructed and filtered images. The scale of polar map was fixed from 0 to 3.2

Typical images, reconstructed by FBP and OSEM, without filtering and with 5, 10, and 15 mm Gaussian filtering are demonstrated in Figure 1. We could confirm that noise increase is associated with an increasing number of iterations; also, noise is decreased and blurring is intensified by increasing Gaussian filter size. We could also obtain the polar map of CFR (Figure 2). A scale of polar map was fixed from 0 to 3.2.

With regard to visual analysis, score 1 was highly prevalent in non-filtered images. The highest score was obtained in images with 10 mm Gaussian filtering. The total scores of non-, 5 mm, 10 mm, and 15 mm filtering were 26, 57, 65, and 47, respectively. Grades 1 and 3 were not counted in 10 mm filtering and non-filtered images, respectively. The grades 1, 2, and 3 were highly counted in non-, 15 mm, and 10 mm filtering, respectively. The IQ was significantly higher by 10 mm Gaussian filtering compared to other filter sizes (*P*<0.001 for both readers). However, no significant difference of IQ was found among FBP and various numbers of iteration in OSEM (*P*=0.923 and 0.855 for readers 1 and 2, respectively).

**Figure 3:**
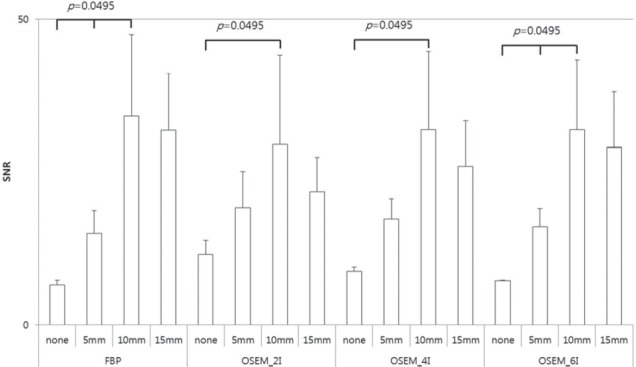
The SNR was calculated in 16 processed images. The highest SNR was calculated in 10 mm Gaussian filter. The SNR of images with 10 mm filtering, reconstructed by FBP and OSEM_6I, was significantly different from the SNR of non-filtered images and images with 5 mm filtering (*P*<0.05), but no significant difference was observed in SNR of images with 15 mm filtering (*P*>0.05). The SNR of images with 10 mm filtering, reconstructed by OSEM_2I and OSEM_6I, showed a significant difference from the SNR of non-filtered image (*P*<0.05); however, it was not significantly different from the SNR of images with 5 mm and 15 mm filtering (*P*>0.05)

SNR was significantly higher in 10 mm Gaussian filter (Figure 3). It was not significantly different in 5 mm (*P*=0.8260), 10 mm (*P*=0.8870), and 15 mm (*P*=0.3996) Gaussian filtering, compared to other reconstruction methods. However, SNR showed a significant difference in non-filtered images (*P*=0.0286), compared to other reconstruction methods. The SNR of images with 10 mm filtering, reconstructed by FBP and OSEM_6I, was significantly different from the SNR of non-filtered images and images with 5 mm filtering (*P*=0.0495); however, it was not significantly different from the SNR of images with 15 mm filtering (*P*=0.8273 and 0.5127).

The SNR of images with 10 mm filtering, reconstructed by OSEM_2I and OSEM_6I, was significantly different from the SNR of non-filtered images (*P*=0.0495); however, it was not significantly different from the SNR of images with 5 mm and 15 mm filtering (*P*>0.05).

The perfusion ratio of normal to abnormal vascular regions which was calculated using the polar map of CFR was not significantly different according to various reconstructed and filtering methods.

## Discussion

In this study, we evaluated various combinations of FBP and OSEM reconstructions and Gaussian filtering methods in ^13^N-NH_3_ myocardial perfusion PET/CT imaging, and found some useful combinations in various settings. If the image quality is poor and performing a quantitative analysis is difficult by FBP without filtering, we recommend any reconstruction method (FBP or other OSEMs) with 10 mm Gaussian filtering. These methods are also helpful in the quantitative measurement of MBF or CFR and making an accurate diagnosis.

Reconstruction is an essential process in cardiac PET imaging. The FBP reconstruction is fast and is considered the most widely used conventional clinical method. In our study, the ranges of rest and stress MBF, as well as CFR, using FBP reconstruction, fit well with the previously reported ranges in various groups of patients (8). Thus, we used MBF and CFR values, measured by FBP reconstruction, as a good reference against which the OSEM method could be compared.

Although OSEM has advantages of noise reduction properties in regions of low uptake (5), it is known that image resolution and noise increase with an increasing number of iterations (6, 7); however, the relative trade-offs for both visualization and measurement tasks are unclear. The selection of the number of iterations is a practical problem in every laboratory, using PET.

**Figure 4:**
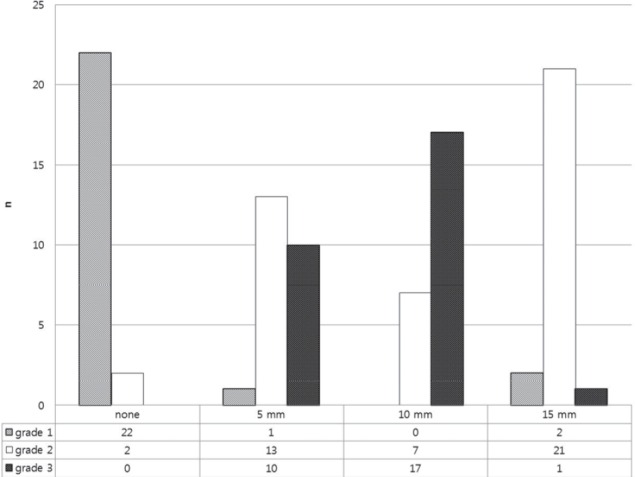
The image quality was visually assessed using a 3-grade scoring system by two independent readers, blinded to the reconstruction methods. The filtering size for 16 stress images was randomly rearranged. The image quality became significantly higher in the 10 mm Gaussian filtering group compared to other filter sizes (*P*<0.001)

Increasing the number of iterations requires more time. For instance, eight OSEM iterations take 10 times longer time than FBP. Moreover, the incremental change is insignificant beyond 10 iterations. We compared the image quality in 2, 4, and 6 OSEM iterations and 5, 10, and 15 mm Gaussian filters, and found that any reconstruction method with 10 mm Gaussian filter showed the best image quality (Figure 4).

**Figure 5:**
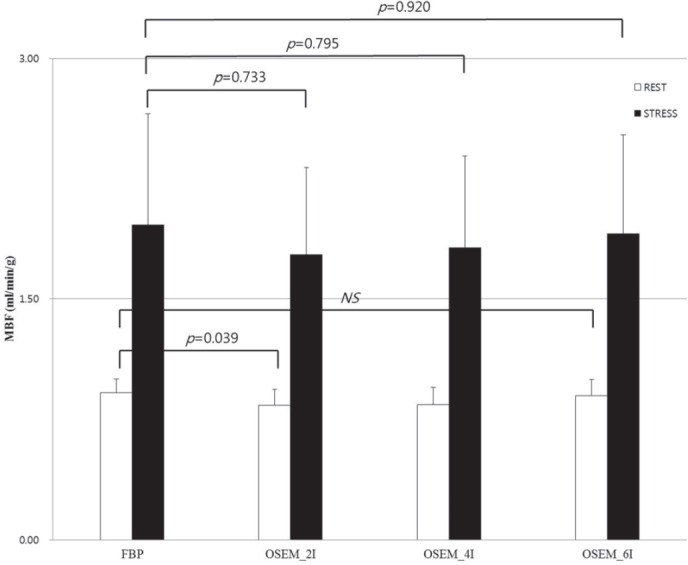
MBFs, filtered by 10 mm Gaussian filter, were compared in each reconstruction method. MBFs at stress were not significantly different; however, for OSEM_2I at rest, MBF was significantly lower than that of FBP

Chen *et al* compared 19 rest/stress image pairs, which were reconstructed by FBP and OSEM with 28 subsets and 2, 6, and 8 iterations (9). Regarding rest MBF, OSEM2, OSEM6, and OSEM8 iterations correlated well with FBP, but OSEM2 caused a significant underestimation of MBF. Considering stress MBF, both OSEM6 and OSEM8 correlated well with the standard FBP method. We tested 2, 4, and 6 OSEM iterations and compared them to FBP. We also found that OSEM 2 with 10 mm Gaussian filter led to a significant (*P*=0.039) underestimation of MBF. OSEM 4 and OSEM 6 showed comparable data to that of FBP (Figure 5). Therefore, we concluded that 4 iterations are most appropriate for daily practice.

Chen *et al* also tested the size of Gaussian filters in the measurement of MBF and CFR (9). They compared 9 image pairs, which were reconstructed by FBP and OSEM with 28 subsets, 8 iterations, and Gaussian 5, 10, or 15 mm postreconstruction smoothing filters. They found that aggressive smoothing with a 15 mm filter caused an underestimation of both rest and stress MBF, compared with FBP. Smoothing with a 10 mm filter caused an underestimation of stress MBF compared to FBP. CFR correlated well among all algorithms, and the calculated values were not significantly different among the algorithms. We also tested Gaussian 5, 10, and 15 mm postreconstruction smoothing filters for the measurement of MBF and CFR. The increasing size of filters resulted in the gradual decrease of MBF, but no change in CFR.

Although ^13^N-NH_3_ cardiac PET/CT imaging is a robust tool to evaluate the pathophysiology of myocardial perfusion, preference of image characteristics and measurement of MBF are not consistent in different laboratories. Reconstruction algorithms do not result in consistent values in each vendor. Optimal protocol may vary from vendor to vendor and from laboratory to laboratory. Therefore, each laboratory should test each algorithm and select an optimal balance between image quality and accurate MBF measurement.

A limitation of this study was the small number of subjects, which did not cover a wide range of coronary artery diseases and healthy cohorts. The assessment of image quality could be affected by the narrow range of disease severity in this study group. However, we compared the results of FBP with various combinations of OSEM iterations and Gaussian filter size; therefore, the statistical power was significant with this number of comparisons.

In conclusion, we recommend finding an optimal reconstruction algorithm in each laboratory in case of poor image quality. In our setting, we found that four OSEM iterations with 10 mm Gaussian filtering showed the best image quality without any change of quantitative values of MBF in ^13^N-NH_3_ myocardial perfusion PET/CT imaging.

## Conclusion

We analyzed images reconstructed by FBP and OSEM methods and filtered by Gaussian filters in the ^13^N-NH_3_ PET and obtained 16 images. The image quality of 16 different images was assessed by signal to noise ratio and visual analysis. We also compared the CFR of these images. Post-reconstruction Gaussian filtering with a filter size of 10 mm significantly enhanced the image quality of [13N]NH3 PET without changing the results of CFR calculation.
